# Glucocerebrosidase expression patterns in the non-human primate brain

**DOI:** 10.1007/s00429-017-1504-1

**Published:** 2017-08-23

**Authors:** Iria G. Dopeso-Reyes, Diego Sucunza, Alberto J. Rico, Diego Pignataro, David Marín-Ramos, Elvira Roda, Ana I. Rodríguez-Pérez, José L. Labandeira-García, José L. Lanciego

**Affiliations:** 1 Basal Ganglia Neuroanatomy Laboratory, Department of Neurosciences, Center for Applied Medical Research (CIMA), Pio XII Avenue 55, Edificio CIMA, 31008 Pamplona, Spain; 2 Centro de Investigación Biomédica en Red sobre Enfermedades Neurodegenerativas (CIBERNED), Madrid, Spain; 3 Laboratory of Neuroanatomy and Experimental Neurology, Department of Morphological Sciences, Faculty of Medicine, University of Santiago de Compostela, Santiago de Compostela, Spain

**Keywords:** Gaucher’s disease, Parkinson’s disease, Substantia nigra, Nucleus basalis of Meynert, Locus ceruleus, Alpha-synuclein, Tau, GCase, GBA1

## Abstract

Glucocerebrosidase (GCase) is a lysosomal enzyme encoded by the GBA1 gene. Mutations in GBA1 gene lead to Gaucher’s disease, the most prevalent lysosomal storage disorder. GBA1 mutations reduce GCase activity, therefore promoting the aggregation of alpha-synuclein, a common neuropathological finding underlying Parkinson’s disease (PD) and dementia with Lewy bodies. However, it is also worth noting that a direct link between GBA1 mutations and alpha-synuclein aggregation indicating cause and effect is still lacking, with limited experimental evidence to date. Bearing in mind that a number of strategies increasing GCase expression for the treatment of PD are currently under development, here we sought to analyze the baseline expression of GCase in the brain of *Macaca fascicularis*, which has often been considered as the gold-standard animal model of PD. Although as with other lysosomal enzymes, GCase is expected to be ubiquitously expressed, here a number of regional variations have been consistently found, together with several specific neurochemical phenotypes expressing very high levels of GCase. In this regard, the most enriched expression of GCase was constantly found in cholinergic neurons from the nucleus basalis of Meynert, dopaminergic cells in the substantia nigra pars compacta, serotoninergic neurons from the raphe nuclei, as well as in noradrenergic neurons located in the locus ceruleus. Moreover, it is also worth noting that moderate levels of expression were also found in a number of areas within the paleocortex and archicortex, such as the entorhinal cortex and the hippocampal formation, respectively.

## Introduction

Glucocerebrosidase (GCase) is a lysosomal enzyme involved in the hydrolysis of the glycosphingolipid glucosylceramide to ceramide and glucose. Homozygotic mutations in the gene coding for GCase (GBA1) cause Gaucher’s disease (GD). Although GD is categorized as a rare disease, it is the most prevalent one within the broad spectrum of lysosomal storage disorders. Up to three different types of GD have been described (GD I–III) based on clinical disease progression and the presence of neurological manifestations (Jmudiak and Futerman 2005; Grabowski 2008).

GCase has currently deserved increased attention in the field of Parkinson’s disease and related synucleinopathies. A first study was published long ago by Van Bogaert and Froelich (1939) who reported about one GD patient that exhibited extra-pyramidal symptoms. Later on, Neurdofer et al. (1996) have found typical parkinsonian symptoms—resting tremor, bradykinesia and rigidity—in a cohort of six GD patients. The presence of a direct link between GBA1 mutations and synucleinopathies such as PD and dementia with Lewy bodies (LBD) has been uncovered by multicenter genetic studies (Sidransky 2005; Sidransky et al. 2009; Goker-Alpan et al. 2004, 2008, 2012; reviewed in Aflaki et al. 2017). These studies appointed GBA1 mutations as the most common genetic risk factor for developing PD and indeed the association between GBA1 mutations and LBD is even stronger than for PD (Nalls et al. 2013). Furthermore, it is also worth noting that after a follow-up of 2 years, both GD patients as well as heterozygous carriers of GBA1 mutations showed prodromal signs of parkinsonism, such as olfactory deficits, higher UPDRS motor scores, lower cognitive assessment scores, REM sleep disturbances and higher depression scores (McNeil et al. 2012; Beavan et al. 2015). Regarding the clinical phenotype, GBA1-associated PD is almost identical to idiopathic PD, besides a slightly earlier disease onset and greater risk for neuropsychiatric symptoms (reviewed in Midgalska-Richards and Schapira 2016; Blanz and Saftig 2016). Moreover, an association between the severity of the PD phenotype and the burden of GBA1 mutations has also been recently reported (Thaler et al. 2017). Finally, it is also worth noting that GBA1 mutations were found in 17% of PD patients being treated with deep brain stimulation (Angeli et al. 2013). Although the overall incidence of PD in GBA1 mutation carriers ranges between 3 and 15% of PD individuals (mainly related to the way in which the GBA1 gene is sequenced), it can be roughly estimated that 10% of PD patients hold a GBA1 mutation (reviewed in Midgalska-Richards and Schapira 2016; Blanz and Saftig 2016; see also Aflaki et al. 2017). While the genetic link between GBA1 mutations and synucleinopathies such as PD and LBD is the strongest argument linking GCase deficit with the appearance of synucleinopathies, the ultimate basis for this association has remained elusive, with very little experimental evidence to date.

While as a lysosomal enzyme, GCase is ubiquitously expressed throughout all organs of the body, also including the brain (http://www.proteinatlas.org), very little is known about the patterns of expression of GCase in the control and diseased brain. Available data are mainly limited to changes in GCase enzymatic activities, whereas neuropathological studies are often restricted to specific brain areas such as the substantia nigra and the hippocampal formation. In this regard, several studies reported reduced GCase activity in the substantia nigra of PD brains (Gegg et al. 2012; Chiasserini et al. 2015). Furthermore, within three GD patients and four heterozygotic carriers, 32–90% of Lewy bodies were found to display GCase immunoreactivity (Goker-Alpan et al. 2010). Finally, strong CGase immunoreactivity was found in the hippocampal formation (regions CA2–4) both in control brains as well as in GD type I patients (Wong et al. 2004). Bearing in mind the current broad interest in glucocerebrosidase as a potential target candidate for the treatment of PD and related synucleinopathies, here we provide a comprehensive mapping of baseline GCase expression levels throughout the entire brain of the long-tailed macaque.

## Materials and methods

Here we have used two series of coronal brain sections available in our macaque brain bank (two series per animal). Sections were taken from three naïve adult male *Macaca fascicularis* primates (body weight 3.4–4.5 kg). Animal handling was conducted in accordance with the European Council Directive 210/63/UE as well as in keeping with the Spanish legislation (RD53/2013). The experimental design was approved by the Ethical Committee for Animal Testing of the University of Navarra (ref: 009-12). All animals were captive-bred and supplied by R. C. Hartelust (Leiden, The Netherlands).

### Perfusion and tissue processing

Animals were anesthetized with an overdose of 10% chloral hydrate and perfused transcardially. The perfusates consisted of a saline Ringer solution followed by 3000 ml of a fixative solution containing 4% paraformaldehyde and 0.1% glutaraldehyde in 0.125 M phosphate buffer (PB), pH 7.4. Perfusion was continued with 1000 of a cryoprotectant solution made of 10% glycerin and 1% dimethyl sulphoxide (DMSO) in 0.125 M PB, pH 7.4. Once the perfusion was completed, the skull was opened and the brain removed and stored for 48 h in a cryoprotective solution containing 20% glycerin and 2% DMSO in 0.125 M PB, pH 7.4. Next, frozen serial coronal sections (40 μm-thick) were obtained on a sliding microtome and collected in 0.125 M PB cryoprotective solution containing 20% glycerin and 2% DMSO, as 10 series of adjacent sections.

### Histological processing

For each of the three macaques, one entire series of rostrocaudal sections ranging from 10 mm rostral to the anterior commissure and 24 mm caudal to the anterior commissure were used for the immunoperoxidase detection of GCase. Individual sections taken from the second series of sections were used for performing multiple immunofluorescent stains combining GCase with the detection of a variety of markers such as choline acetyltransferase (ChAT), tyrosine hydroxylase (TH) and serotonin (5HT).

For the immunoperoxidase detection of GCase, free-floating sections were rinsed with Tris buffer pH 7.4 (TBS) and then incubated for 40 min with a 0.3% solution of H_2_O_2_ in methanol to block the endogenous peroxidase activity. After several rinses in the TBS solution, the sections were incubated in a blocking solution containing 1% cold fish gelatin (Sigma), 1% bovine serum albumin (BSA; Sigma) and 0.05% Triton X-100 (Sigma) in TBS (TBS-Tx) for 1 h, followed by an overnight incubation with a monoclonal mouse anti-GBA antibody (1:500; Abcam, ref: ab55080). After several rinses in TBS-Tx, sections were incubated for 30 min with a biotinylated donkey anti-mouse IgG (1:600; Jackson Laboratories, ref: 715-066-150). Sections were next rinsed several times in TBS-Tx and further incubated for 30 min in an ABC solution (Vectastain ABC HRP kit; Vector Laboratories, ref: PK4000). Specificity of the anti-GBA antibody was previously shown by Barneveld et al. (1983). Additional negative controls were performed by removal of the primary antibody, a procedure resulting in complete lack of stain. Delineation of the boundaries of brain nuclei showing GCase labeling and nomenclature was based on the atlases of Lanciego and Vázquez (2012) and Martin and Bowden (1996, 1997).

A similar procedure was conducted for the immunofluorescent detection of GCase combined with a number of neuronal markers. In this case, the following primary antibodies were used: mouse anti-GBA (1:500; Abcam), goat anti-TH (1:50; Santa Cruz, ref: sc-7847), goat anti-ChAT (1:100; Millipore, ref: AB114P), and rabbit anti-5HT (1:5000; immunostar, ref: 20080). Detection was carried out using the following secondary antibodies (all diluted 1:200 and incubated for 2 h at room temperature): Alexa Fluor^®^ 488-conjugated donkey anti-mouse IgG (Molecular Probes-Invitrogen, ref: A21202), Alexa Fluor^®^ 546-conjugated donkey anti-goat IgG (Molecular Probes-Invitrogen, ref: A11056), and an Alexa Fluor^®^ 555-conjugated donkey anti-rabbit IgG (Molecular Probes-Invitrogen, ref: A31572). Sections were incubated in a solution of Topro-3 for counterstaining purposes (1:400, 1 h at room temperature; Invitrogen, ref: T3605).

Sections were finally rinsed in TBS and mounted on SuperFrost Ultra Plus slides, dried at room temperature and coverslipped with DePex^®^ (VWR International).

Immunoperoxidase sections were inspected and photographed with a Nikon Eclipse 800 brightfield microscope. GCase expression levels were evaluated by four independent neuroanatomists according to a scoring scale ranging from 1 to 4 (reflecting low to high intensity, respectively). Obtained scores were averaged to finally generate an overall pattern of GCase staining intensities across all inspected brain areas and nuclei. Sections processed for immunofluorescence were inspected under a confocal laser-scanning microscope (LSM 800, Zeiss, Germany). To ensure appropriate visualization of the labeled elements and to avoid false positive results, the emission from the argon laser at 488 nm was filtered through a band pass filter of 505–530 nm and color-coded in green. The emission following excitation with the helium laser at 543 was filtered through a band pass filter of 560–615 nm and color-coded in red. Finally, a long-pass filter of 650 nm was used to visualize the emission from the helium laser at 633 and color-coded in dark blue.

## Results

The immunohistochemical detection of GCase was carried out in serial coronal sections comprising the entire rostrocaudal extent of the brain in the long-tailed macaque, *Macaca fascicularis*. As a lysosomal enzyme, GCase expression was found to be expressed throughout all inspected brain cortical areas and subcortical structures. However, region-specific differences were consistently observed, comprising changes in baseline expression levels between neocortex, archicortex and paleocortex, as well as within subcortical territories as the amygdala, hypothalamus and the caudal intralaminar nuclei. Most importantly, neurons giving rise to diffuse ascending systems were the ones that exhibited by far the highest GCase expression levels.

### Cerebral cortex

**Fig. 1:**
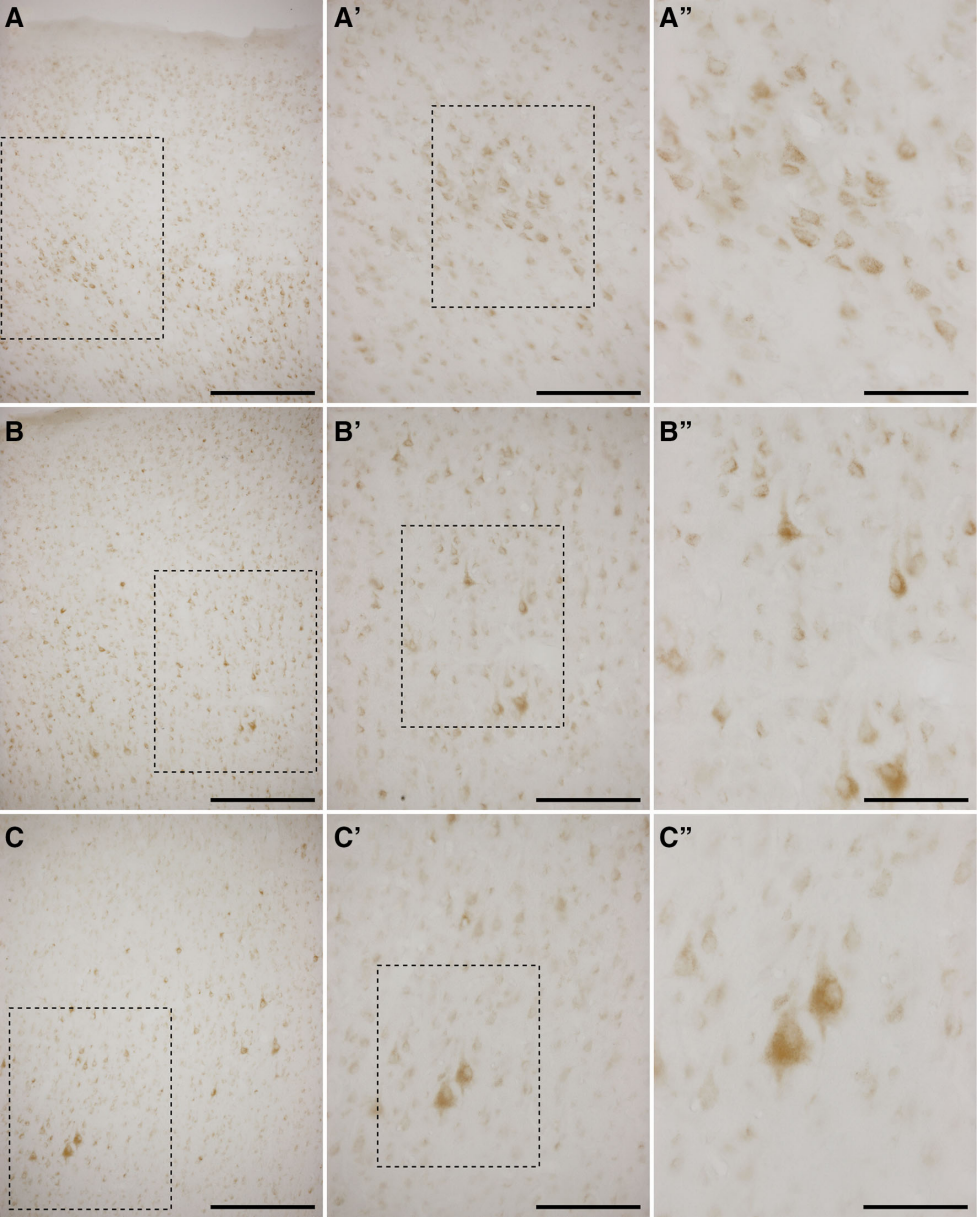
Immunohistochemical detection of GCase in neocortical areas. Representative images taken at different magnifications from the anterior cingulated gyrus (**a**–**a″**), superior frontal gyrus (**b**–**b″**) and inferior frontal gyrus (**c**–**c″**). All labeled cells displayed a weak GCase immunoreactivity. GCase stain was slightly more prominent in pyramidal cell layers. Giant pyramidal cells (Betz neurons) are the ones more easily appreciated. *Scale bar* is 300 μm in **a**–**c**; 150 μm in **a′**–**c′**; and 75 μm in **a″**–**c″**

**Fig. 2:**
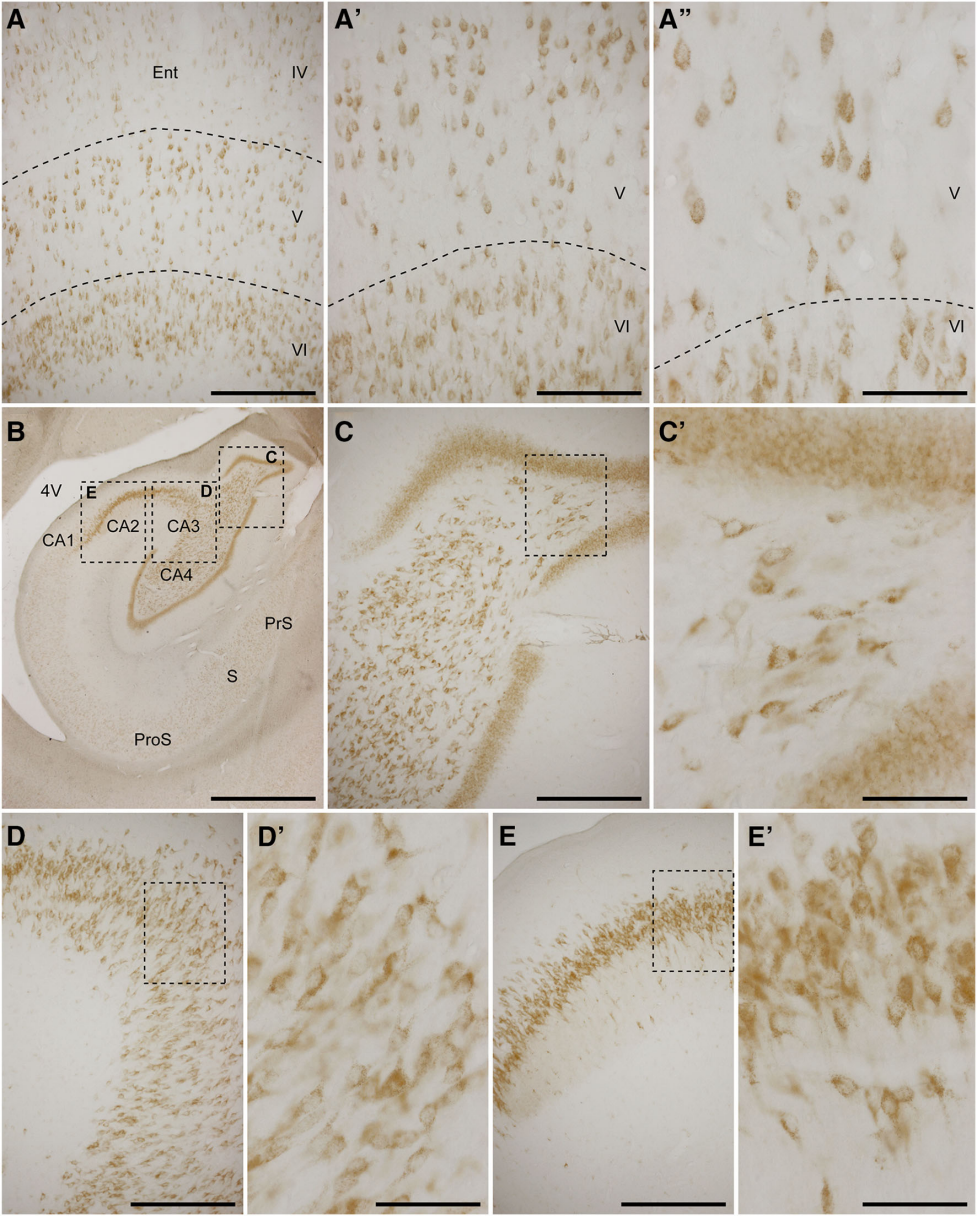
Immunohistochemcial detection of GCase in archicortex and paleocortex. Representative images taken at different magnifications from the entorhinal cortex (**a**–**a″**) and the hippocampal formation (**b**–**e′**). Deep layers of the entorhinal cortex (lamina V and VI) displayed a moderate GCase staining. At the level of the hippocampal formation, fields CA2–4 are the territories showing a more intense GCase immunoreactivity. By contrast, CA1 field, prosubiculum (*ProS*), subiculum (*S*) and presubiculum (*PreS*) only showed a very weak staining. *Scale bar* is 300 μm in **a**; 150 μm in **a′**; 75 μm in **a″**; 1500 μm in **b**, 300 μm in **c**–**e**; and 75 μm in **c**–**e′**

**Fig. 3:**
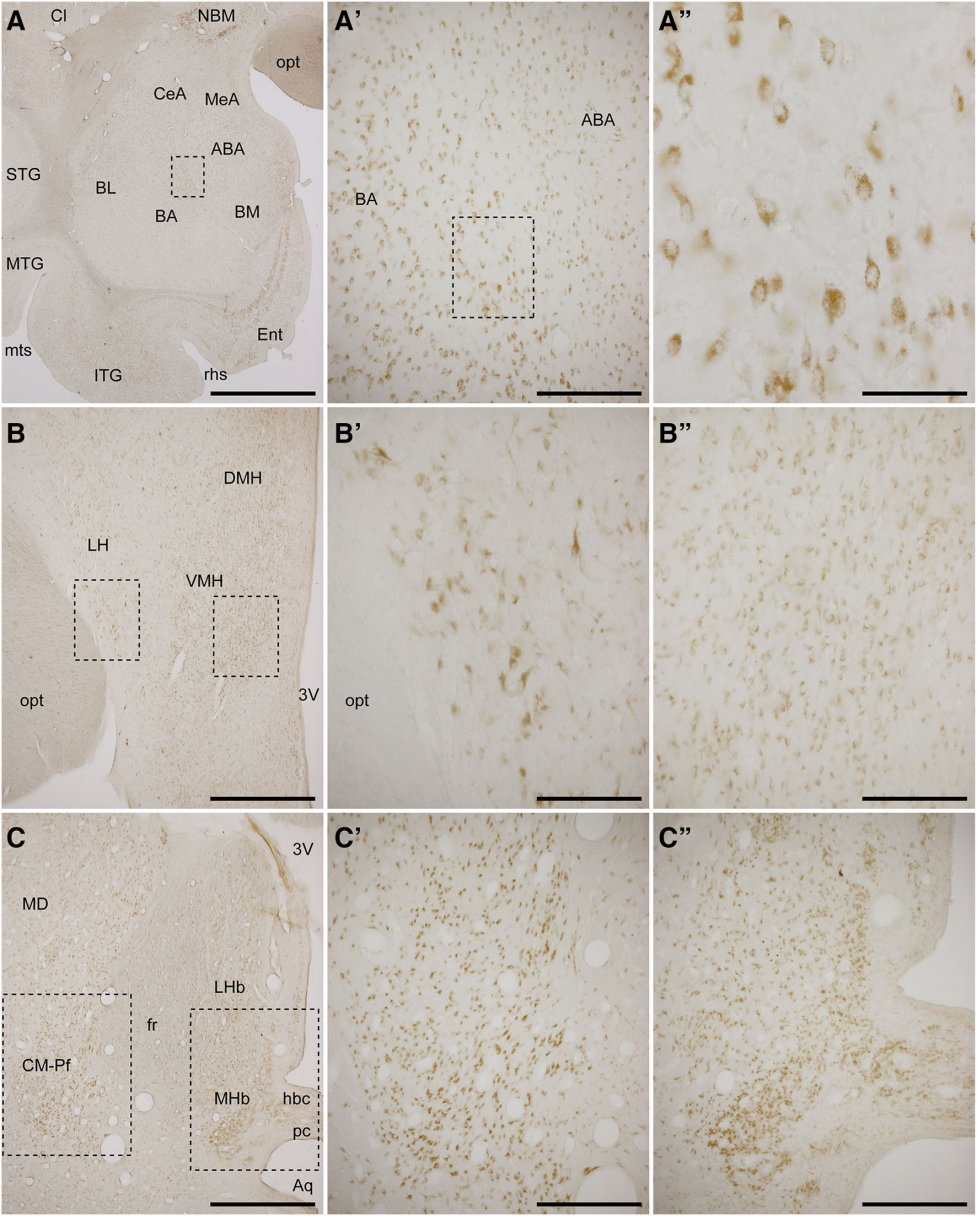
Immunohistochemical detection of GCase in a number of subcortical structures. Representative images taken at different magnifications from the amygdaloid complex (**a**–**a″**), the hypothalamus (**b**–**b″**) and the caudal thalamus (**c**–**c″**). At the level of the amygdaloid complex, the basal amygdaloid nucleus (*BA*) exhibited a higher staining intensity than any other nuclei of the amygdaloid complex. Regarding the hypothalamus, the lateral hypothalamic area (*LH*) showed a more intense GCase immunoreactivity than the dorsomedial (*DMH*) and ventromedial (*VMH*) hypothalamic nuclei. The centromedian–parafascicular thalamic complex (*CM–Pf*) is the thalamic nucleus with higher GCase stain, whereas for the habenular complex, the medial habenular nucleus (*MHb*) is more intensely stained than the lateral habenular nucleus (*LHb*). *opt* optic tract, *NBM* nucleus basalis of Meynert, *Cl* claustrum, *STG* superior temporal gyrus, *MTG* medial temporal gyrus, *ITG* inferior temporal gyrus, *Ent* entorhinal cortex, *mts* middle temporal sulcus, *rhs* rhinal sulcus, *ABA* accessory basal amygdaloid nucleus, *BA* basal amygdaloid nucleus, *BL* basolateral amygdaloid nucleus, *BL* basolateral nuclear group, *CeA* central amygdaloid nucleus, *MeA* medial amygdaloid nucleus, *3V* third ventricle, *MD* mediodorsal thalamic nucleus, *pc* posterior commissure, *Aq* aqueduct, *hbc* habenular commissure. *Scale bar* is 3000 μm in **a**; 150 μm in **a′**, **b′**, **b″**; 120 μm in **b**, **c**; 300 μm in **c′**, **c″**; and 75 μm in **a″**

Neurons from all neocortical areas including the frontal, parietal, occipital and temporal lobes exhibited a weak GCase immunoreactivity. Pyramidal neurons within layers III, V and VI are the ones showing slightly higher expression levels, particularly giant layer V pyramidal neurons from the frontal lobe (Fig. 1). By contrast, neurons located in older brain cortices such as the archicortex (entorhinal cortex) and paleocortex (hippocampal formation) displayed a more robust GCase immunoreactivity. Within the entorhinal cortex, a tri-layered pattern was typically observed; best exemplified by layers V and VI (Fig. 2a–a″; see also Fig. 3a). Furthermore and in keeping with has been reported in human brains (Wong et al. 2004), strong GCase staining was found in hippocampal regions CA2–4, whereas in the CA1 region very low expression levels were constantly found (Fig. 2b–e′). This also applies to hippocampal-related territories such as the prosubiculum, subiculum and presubiculum, all of them showing very sparse labeling (Fig. 2b).

### Subcortical structures

The patterns of GCase expression were also analyzed in a number of subcortical structures, these comprising the basal ganglia nuclei, claustrum, septum, amygdala, hypothalamus, thalamus and brainstem. While a ubiquitous and weak GCase immunoreactivity was found within all these structures, neurons within several nuclei exhibited more intense GCase expression levels. For instance, at the level of the amygdaloid complex, the basal amygdaloid nucleus is more intensely stained than any other amygdaloid nuclei (Fig. 3a–a″). This is also the case for the lateral hypothalamus when compared with the ventral and dorsal medial hypothalamic nuclei, the latter two showing a sparser staining (Fig. 3b–b″). Furthermore, within the septal nuclei, only the major island of Calleja showed a moderate neuropil staining. At the thalamic level, the highest GCase expression levels were found in the caudal intralaminar nuclei (centromedian–parafascicular complex, see Fig. 3c, c′). Some minor differences were also found in the habenular complex. When comparing lateral and medial habenular nuclei, the latter showed a more intense expression pattern (Fig. 3c, c″). Basal ganglia-related nuclei such as the caudate and putamen nuclei, both segments of the globus pallidus, the subthalamic nucleus and the substantia nigra pars reticulata displayed a very weak GCase staining, with the only exception of the large cholinergic striatal interneurons that exhibited very intense GCase expression levels (Fig. 5e–h′). For all the remaining structures inspected, ranging from the red nucleus in the mesencephalon to the inferior olive of the brainstem—also including cerebellar cortex and deep cerebellar nuclei—only a very weak pattern of GCase immunoreactivity was consistently found, without any noticeable difference between different nuclei.

### Diffuse ascending systems

**Fig. 4:**
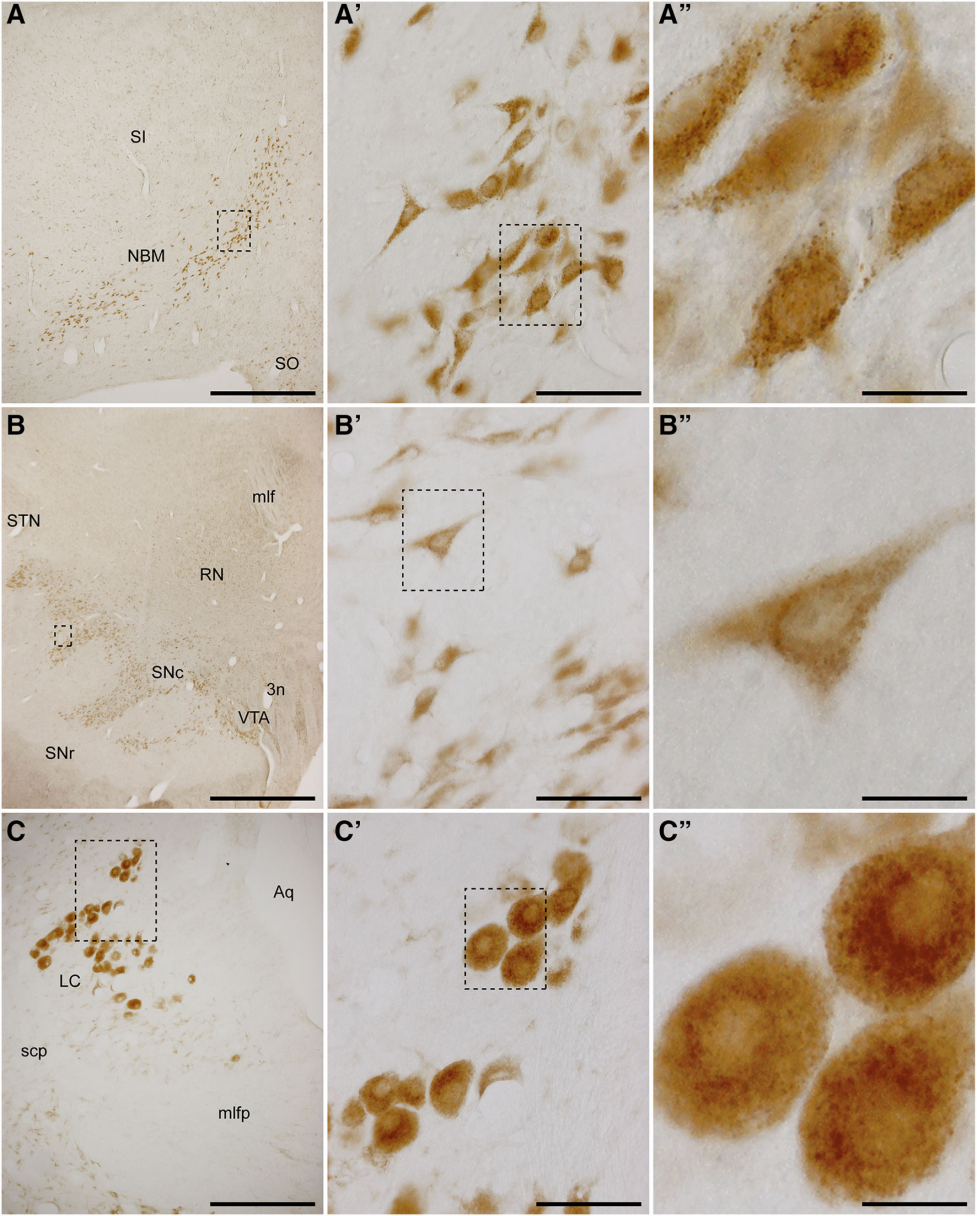
Immunohistochemical detection of GCase in neuronal groups giving rise to diffuse ascending systems. Cholinergic neurons from the nucleus basalis of Meynert (**a**–**a″**), dopaminergic neurons from the substantia nigra pars compacta (**b**–**b″**) and noradrenergic neurons from the locus ceruleus (**c**–**c″**) were the ones showing the highest GCase staining intensities throughout the entire macaque brain. All these neurons typically exhibited intense granular-like GCase immunoreactivity. *SI* substantia innominata, *SO* supraoptic nucleus, *NBM* nucleus basalis of Meynert, *mlf* medial longitudinal fasciculus, *RN* red nucleus, *STN* subthalamic nucleus, *SNc* substantia nigra pars compacta, *SNr* substantia nigra pars reticulata, *VTA* ventral tegmental area, *3n* third cranial nerve, *Aq* aqueduct, *LC* locus ceruleus, *scp* superior cerebellar peduncle, *mlfp* medial longitudinal fasciculus of pons. *Scale bar* is 750 μm in **a**; 1500 μm in **b**; 300 μm in **c**; 75 μm in **a′**, **b′**, **c′**; and 18.75 μm in **a″**, **b″**, **c″**

**Fig. 5:**
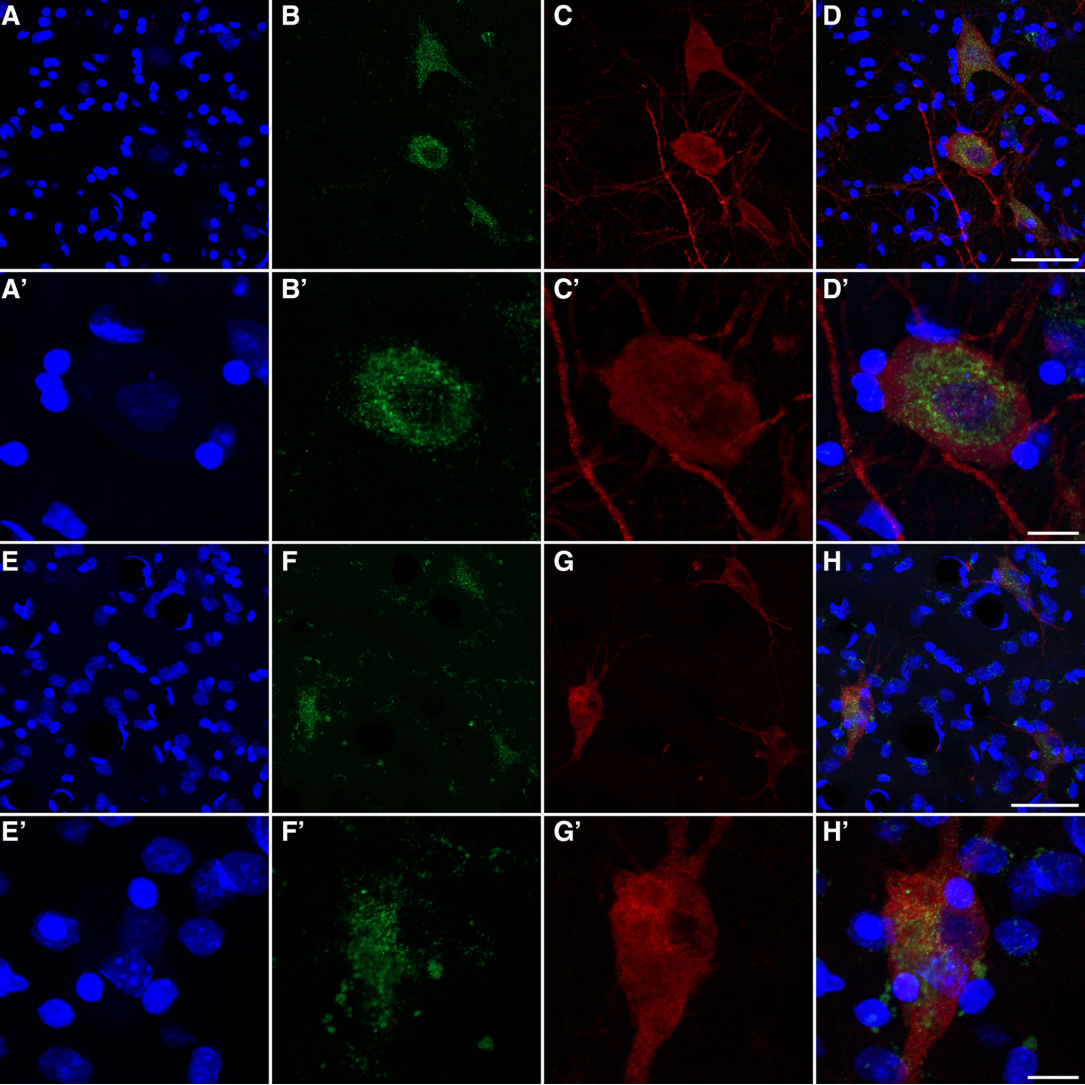
Cholinergic neurons and GCase expression. Multiple immunofluorescent stains showing the expression of GCase within cholinergic neurons from the nucleus basalis of Meynert (**a**–**d′**) as well as in striatal cholinergic interneurons from the putamen (**e**–**h′**). GCase expression is shown in the *green channel*; ChAT-immunoreactivity in the *red channel*. Sections were counterstained with Topro-3 as illustrated in the *blue channel*. *Scale bar* is 40 μm in **a**–**d** and **e**–**h**, and 10 μm in **a′**–**d′** and **e′**–**h′**

**Fig. 6:**
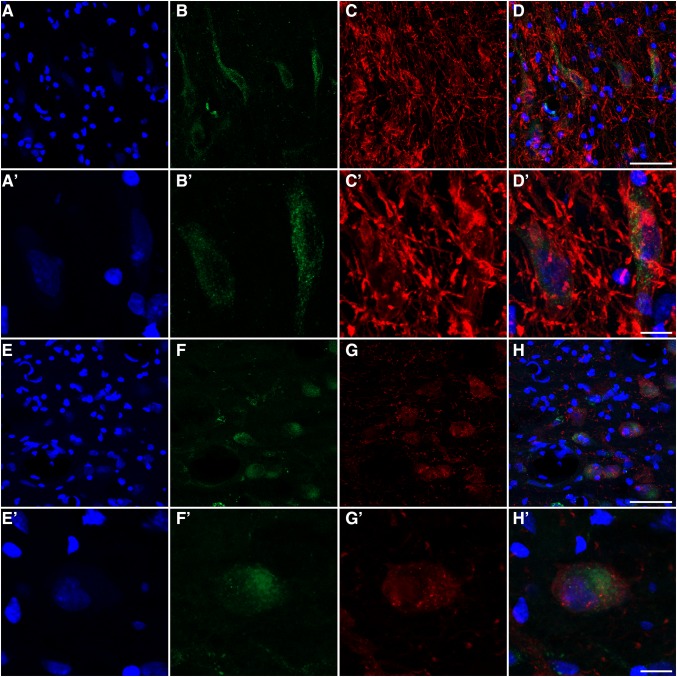
GCase expression in dopaminergic and serotoninergic neurons. Multiple immunofluorescent stains showing the GCase expression within dopaminergic neurons from the substantia nigra pars compacta (**a**–**d′**) as well as in serotoninergic neurons from the dorsal raphe nuclei (**e**–**h′**). *Green channel* shows the GCase expression, whereas tyrosine-positive neurons or serotoninergic neurons are illustrated in the *red channel* (**c**, **c′**, **g**, **g′**, respectively). Sections were counterstained with Topro-3 as seen in the *blue channel*. *Scale bar* is 40 μm in **a**–**d** and **e**–**h**, and 10 μm in **a′**–**d′** and **e′**–**h′**

**Fig. 7:**
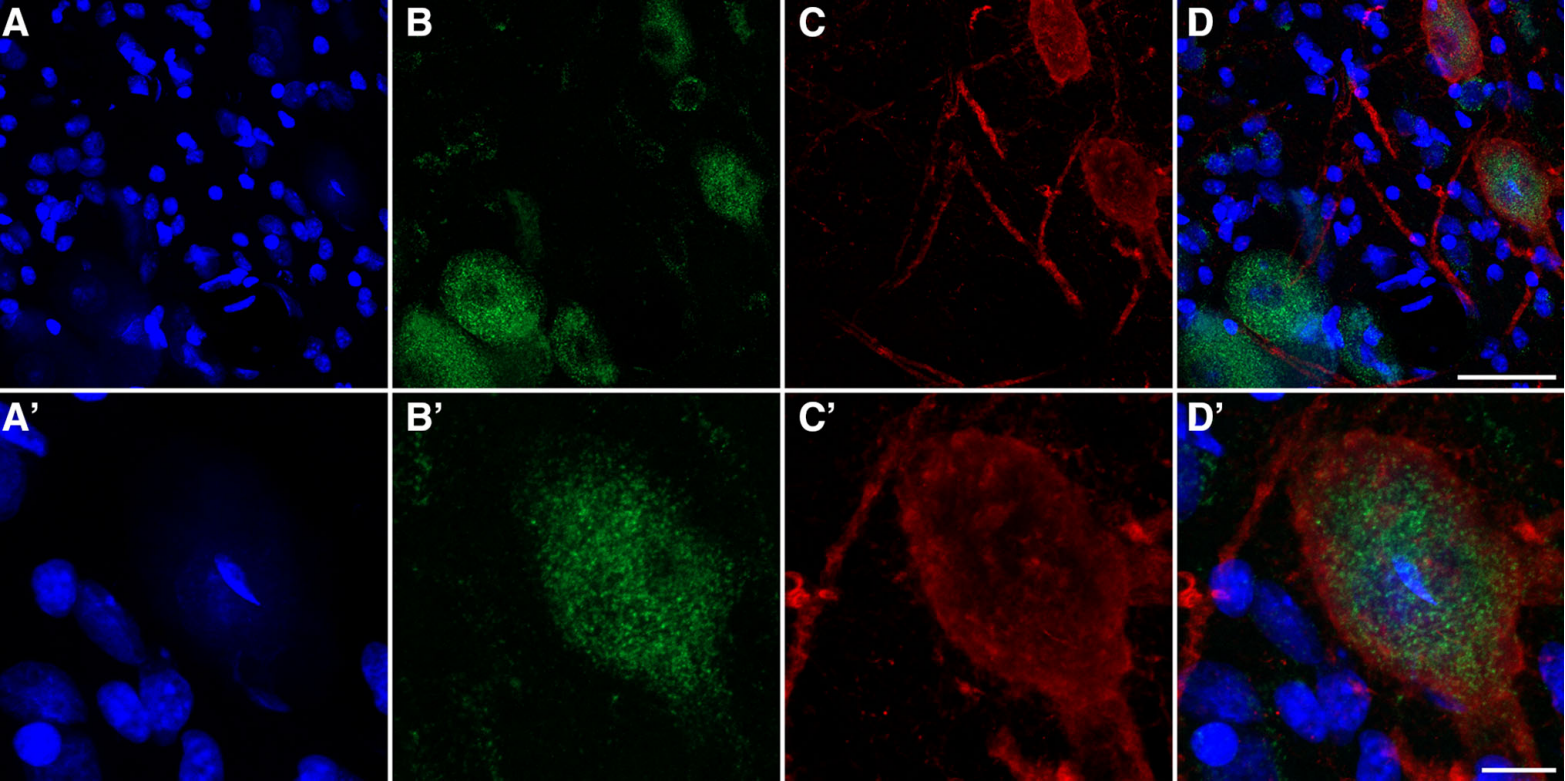
GCase expression levels in the locus ceruleus. *Green channel* shows the GCase expression whereas tyrosine-positive neurons are illustrated in the *red channel*. Sections were counterstained with Topro-3 as seen in the *blue channel*. *Scale bar* is 40 μm in **a**–**d**, and 10 μm in **a′**–**d′**

Besides the overall weak or moderate GCase expression levels observed throughout the entire rostrocaudal extension of the non-human primate brain, very intense expression levels were characteristically found in neurons giving rise to the so-called “diffuse ascending systems” (Thierry et al. 1990; see Fig. 4). These are very small nuclei or cellular groups found in restricted locations providing the brain with specific neurotransmitters such as acetylcholine (cholinergic neurons from the nucleus basalis of Meynert), dopamine (substantia nigra pars compacta), serotonin (raphe nuclei) and noradrenaline (locus ceruleus). Within all these cellular groups, the intensity of GCase staining is so high that it is even visible to the naked eye, i.e., without the need of a microscope. In non-human primates, the nucleus basalis of Meynert (NBM) is made up of a thin band of densely packed cholinergic neurons located in the forebrain just below the ventral pallidum/substantia innominata. These neurons exhibited very intense GCase expression levels with a typical granular-like appearance, distributed throughout the cytoplasm (Fig. 4a–a″). Similar GCase staining patterns were also found in the substantia nigra pars compacta and in the locus ceruleus (Fig. 4b–b″, c–c″, respectively). The cholinergic, dopaminergic, serotoninergic and noradrenergic identities of these neurons expressing such a very high GCase immunoreactivity was confirmed by immunofluorescent studies, as shown in Figs. 5, 6 and 7.

## Discussion

**Fig. 8:**
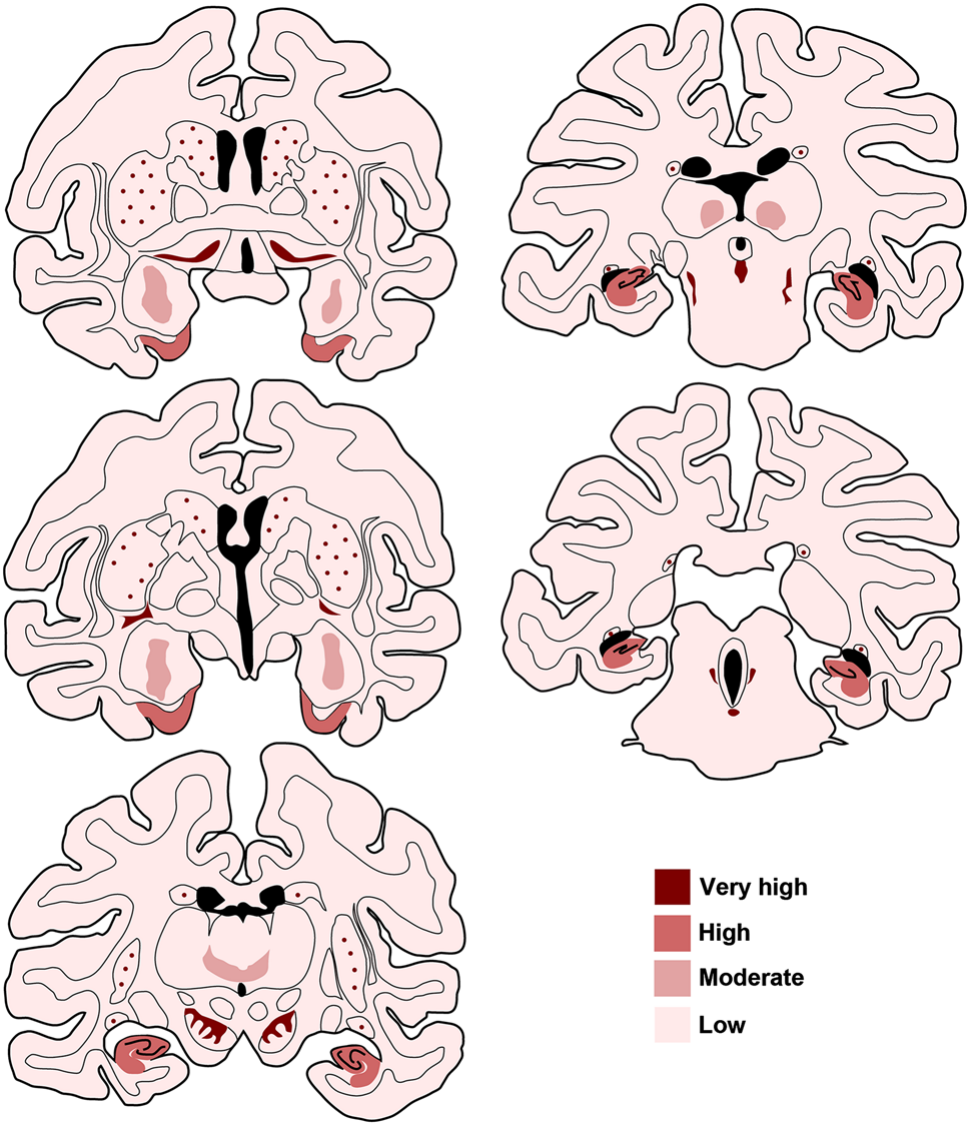
Schematic diagram summarizing the observed GCase staining patterns throughout different rostrocaudal brain sections of the non-human primate brain. The staining intensities are coded according to *different colored gradients*. Striatal cholinergic neurons as well as neurons giving rise to diffuse ascending systems are by far the ones showing the highest GCase staining intensities. High levels of GCase immunoreactivity were also found in the hippocampal formation and in the entorhinal cortex

While the data provided here are consistent with a ubiquitous distribution of GCase in the non-human primate brain, a number of regional variations in the GCase expression levels were constantly found. For instance, older brain cortices such as the archicortex and the paleocortex showed a more intense immunoreactivity than any neocortical area. Most importantly, neurons giving rise to diffuse ascending systems were the ones showing the highest levels of GCase immunoreactivity. The overall pattern of GCase immunoreactivity in the non-human primate brain is summarized in Fig. 8.

At present it is broadly accepted that mutations in the gene coding for GCase (GBA1 gene) represent the main genetic risk factor for the development of PD and LBD. Although mutations in genes such as LRRK2, PINK1 and DJ-1 have often been implicated in the pathophysiology of synucleinopathies, GBA1 mutations are the ones showing the highest prevalence (Neumann et al. 2009). While GCase deficit and alpha-synuclein aggregation are apparently directly linked to each other, the ultimate mechanisms sustaining this association still remain elusive. Indeed, it may well be the case that instead of existing a linear relationship between GCase and alpha-synuclein, GCase loss-of-function may trigger a number of changes in lipid metabolism, autophagy, mitochondrial function and dysfunction, ER stress and cytotoxicity that all together would be the actors truly sustaining the aggregation of misfolded alpha-synuclein (Wong et al. 2004; Mazzuli et al. 2011; Kurawa-Akanbi et al. 2012; Murphy et al. 2014; Chiasserini et al. 2015; Gegg and Schapira 2016). Data gathered from PD patients showed that GCase protein levels and enzymatic activities are reduced in brain areas showing alpha-synuclein aggregation (Murphy et al. 2014). GCase mRNA levels were significantly reduced in the substantia nigra in PD and LBD patients (Chiasserini et al. 2015). These findings are in full keeping with earlier reports showing a substantial decrease on GCase activity in a number of brain areas, from which the greatest deficiency was found in the substantia nigra (Gegg et al. 2012). In summary, it seems that GCase and alpha-synuclein are forming together a bidirectional, vicious circle that self-perpetuates disease progression (Mazzuli et al. 2011). These evidences have paved the way for the development of disease-modifying therapies intended to increase GCase levels in an attempt to slow-down or ideally revert the progressive aggregation of alpha-synuclein. In this regard, it has been recently shown that the co-injection of adeno-associated viral vectors coding for GBA1 and mutated forms of alpha-synuclein in rats fully prevented nigral dopaminergic neurons from neurodegeneration with a follow-up of 6 months (Rocha et al. 2015). Besides gene therapies, there currently is strong interest from pharmaceutical companies for the development of different approaches such as chaperones, allosteric modulators and BBB-passing enzyme replacement therapies, to mention just a few, all these approaches sharing a common rationale of increasing brain levels of GCase (reviewed in Kelly et al. 2017; see also Sardi et al. 2013). Indeed, it has been recently shown that the inhibition of glucosylceramide synthase in a mice model of Gaucher-related synucleinopathy efficiently reduces the levels of alpha-synuclein, ubiquitin and Tau aggregates at the level of the hippocampal formation (Sardi et al. 2017).

Finally, it is worth noting that the findings reported here demonstrate that the most enriched GCase levels were consistently found in neurons giving rise to diffuse ascending systems. All these specific neuronal groups share a common engagement in dealing with several distinct types of misfolded proteins such as alpha-synuclein and Tau. For instance, the presence of Lewy body-like pathology in the locus ceruleus is a phenomenon appearing even before the presence of alpha-synuclein aggregates in the substantia nigra (Del Tredici et al. 2002; Braak et al. 2003). Furthermore, it is also known that neurons in the locus ceruleus and substantia nigra pars compacta co-aggregate both alpha-synuclein and Tau proteins in patients suffering from progressive supranuclear palsy (Erro Aguirre et al. 2015). Considering Alzheimer’s disease, formation of abnormally phosphorylated Tau protein was first detected in subcortical nuclei projecting to the cerebral cortex, i.e., the nucleus basalis of Meynert and the locus ceruleus (Braak et al. 2011).
